# Supplementary material for: UPLC-Q-TOF/MS Based Plasma Metabolomics for Identification of Paeonol’s Metabolic Target in Endometriosis
Source: Molecules. 2023 Jan 9;28(2):653. doi: 10.3390/molecules28020653 (PMC9864815; doi:10.3390/molecules28020653)
Supplement: Supplementary file 1 [file molecules-28-00653-s001.zip › molecules-2090424-supplementary.pdf]

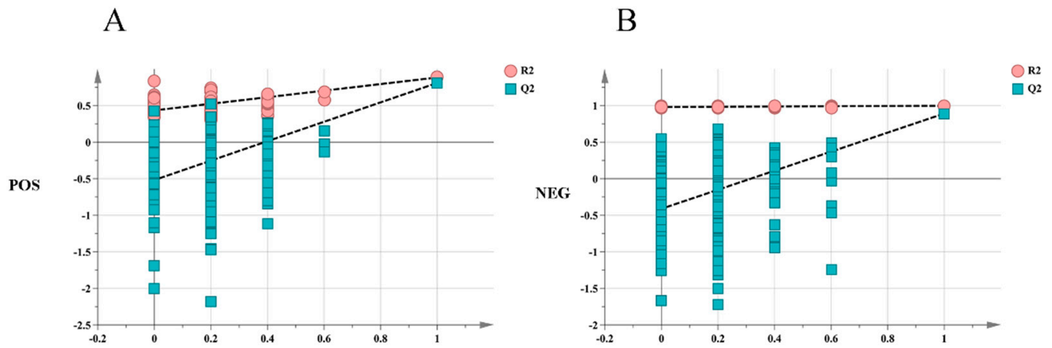

Figure S1. Permutation analysis of OPLS-DA. (A). Statistical validation of the OPLS-DA model using permutation analysis in positive ion mode. **(B).** Statistical validation of the OPLS-DA model using permutation analysis in positive ion mode in negative ion mode.

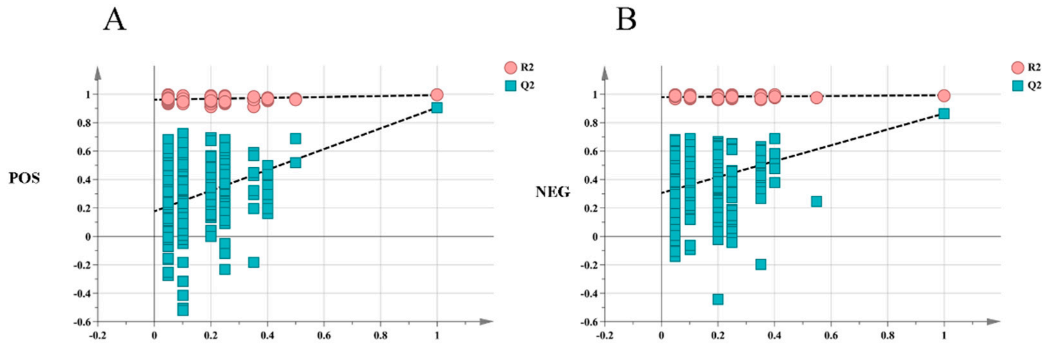

Figure S2. Permutation analysis of PLS-DA. (A). Statistical validation of the PLS-DA model using permutation analysis in positive ion mode. **(B).** Statistical validation of the PLS-DA model using permutation analysis in positive ion mode in negative ion mode.

Table S1. MS/MS information of blood potential biomarkers scanned by positive and negative mode.

No.	Rt min	[M−H]−/[M+H]+	Proposed Composition	Postulated Identity	MS/MS fragment ion (m/z)
1	1.36	[M+H]+	$C_9H_{11}NO_2$	L-Phenylalanine	166.0869[M+H]+, 151.0489[M+H-NH]+, 120.0823[M+H-CH_2O_2]+
2	1.73	[M−H]−	$C_{11}H_{12}N_2O_2$	L-Tryptophan	203.08327[M-H]-, 188.9841[M-H-H_2N]-, 160.0454[M-H-CHO_2]-, 130.0694[M-H-$C_2H_5NO_2$]-, 116.0563[M-H-$C_3H_7NO_2$]-
3	2.20	[M−H]−	$C_9H_9NO_3$	Hippuric acid	178.0504[M-H]-, 162.0572[M-H-O]-, 136.0478[M-H-CO_2]-, 121.1302[M-H-C_2HO_2]-
4	4.79	[M−H]−	$C_{26}H_{46}NO_7P$	LysoPC(18:4(6Z,9Z,12Z,15Z))	514.293[M-H]-, 425.2906[M-H-$C_3H_7NO_2$]-, 407.2827[M-H-C_7H_7O]-, 331.1941[M-H-$C_6H_{18}NO_3P$]-
5	5.51	[M+H]+	$C_{24}H_{36}O_3$	Cervonoyl ethanolamide	373.2745[M+H]+, 319.2409[M+H-C_4H_6]+, 184.0763[M+H-$C_{14}H_{21}$]+, 147.1204[M+H-$C_{13}H_{22}O_3$]+
6	7.25	[M+H]+	$C_{24}H_{48}NO_7P$	LysoPC(16:1(9Z))	494.3242[M+H]+, 311.2596[M+H-$C_5H_{14}NO_4P$]+, 258.1153[M+H-$C_{13}H_{34}NO_2$]+, 184.0742[M+H-$C_{19}H_{36}NO_2$]+
7	7.54	[M+H]+	$C_{26}H_{50}NO_7P$	LysoPC(18:2(9Z,12Z))	520.34[M+H]+, 483.2494[M+H-H_5O_2]+, 337.2709[M+H-$C_5H_{14}NO_4P$]+, 303.0184[M+H-$C_{13}H_{31}NO$]+, 184.0740[M+H-$C_{21}H_{38}NO_2$]+
8	7.71	[M−H]−	$C_{25}H_{44}NO_7P$	LysoPE(0:0/20:4(8Z,11Z,14Z,17Z))	500.279[M-H]-, 325.2380[M-H-$C_6H_{10}NO_3P$]-, 279.2303[M-H-$C_7H_{12}NO_5P$]-, 224.0699[M-H-$C_{18}H_{28}O_2$]-
9	8.13	[M+H]+	$C_{21}H_{44}NO_7P$	LysoPE(16:0/0:0)	454.2939[M+H]+, 362.2845[M+H-$C_2H_6NO_3$]+, 282.2861[M+H-$C_2H_7NO_6P$]+, 184.0746[M+H-$C_{16}H_{32}NO_2$]+

No.	Rt min	[M–H]– /[M+H]+	Proposed Composition	Postulated Identity	MS/MS fragment ion (m/z)
10	8.23	[M+H]+	$C_{24}H_{50}NO_7P$	LysoPC(16:0)	496.3404[M+H]+, 478.3301[M+H-H_2O]+, 313.2761[M+H-$C_5H_{14}NO_4P$]+, 258.1119[M+H-$C_{10}H_{25}NO_3P$]+, 184.0699[M+H-$C_{14}H_{35}NO_4P$]+
11	8.64	[M+H]+	$C_{26}H_{52}NO_7P$	LysoPC(18:1(9Z))	522.3563[M+H]+, 480.3441[M+H-C_3H_6]+, 339.2904[M+H-$C_6H_{18}NO_3P$]+, 258.1117[M+H-$C_{14}H_{34}NO_3$]+, 184.0680[M+H-$C_{16}H_{37}NO_4P$]+
12	8.99	[M–H]–	$C_{20}H_{32}O_3$	5-HETE	319.2278[M-H]-, 269.2482[M-H-CH_6O_2]-, 222.0830[M-H-C_7H_{13}]-, 179.1047[M-H-$C_9H_{16}O$]-
13	9.61	[M+H]+	$C_{23}H_{48}NO_7P$	LysoPC(15:0)	428.0325[M+H]+, 361.2827[M+H-$C_5H_{13}O_3$]+, 184.0758[M+H-$C_{13}H_{33}NO_4P$]+, 135.0766[M+H-$C_{19}H_{41}NO_4$]+
